# The migration pattern of the Charnley femoral stem: a five-year follow-up RSA study in a well-functioning patient group

**DOI:** 10.1007/s10195-012-0187-x

**Published:** 2012-05-11

**Authors:** Kristin Haugan, Otto S. Husby, Jomar Klaksvik, Olav A. Foss

**Affiliations:** 1 Orthopaedic Research Centre, Trondheim University Hospital, 7006 Trondheim, Norway; 2 Orthopaedic Department, Trondheim University Hospital, 7006 Trondheim, Norway

**Keywords:** RSA, Charnley, THR, Five-year follow-up, Migration pattern

## Abstract

**Background:**

Implant stability is considered vital to long-time implant survival in total hip arthroplasty (THA), since loose implants are reported to be a major cause of hip revision. There is an association between early implant micromotion and increased risk of revision. More implant-specific data are needed to establish acceptable levels of early implant movement.

**Materials and methods:**

Thirty-five patients (36 hips) undergoing Charnley THA were followed with repeated clinical, radiographic, and radiostereometric analysis (RSA) over 5 years. Twenty-three patients attended 5 years postoperatively.

**Results:**

The patient group was well functioning based on the radiological and clinical evaluations. The stems constantly moved up to 5 years postoperatively, with subsidence, retroversion, and varus tilt, based on the RSA.

**Conclusion:**

Continuous movement of the Charnley stem was observed up to 5 years postoperatively in a well-functioning patient group. The migration data presented herein could be useful when defining acceptable migration limits for certain types of cemented femoral stems.

## Introduction

Implant stability influences the revision rate observed in total hip arthroplasty (THA). According to the Swedish, Australian and Norwegian hip registers, aseptic loosening is the most common cause of revision [1–3]. Revision surgery is expensive to both patients and the community. Major efforts have been made to reduce these numbers from the earliest days of THA.

Radiostereometric analysis (RSA) has become a gold standard when measuring implant migrations with respect to the cement mantle as well as the surrounding bone [4]. RSA can describe early implant migration, and is therefore well suited and recommended when introducing new implants into clinical use and minimizing the number of enrolled patients [5].

The main rationale for monitoring early implant migration is based on results from clinical studies reporting an association between early subsidence and increased risk of early or mid-term hip implant revision. Early migration [6], continuous migration [7], and subsidence in combination with medial and posterior migration [6] are of concern. However, when defining acceptable limits for migration, factors such as measurement procedures, implant design, and fixation principles should be taken into account.

The level of early implant stability is mainly determined by the fixation method used. In cement fixation of femoral stems, two different principles apply [8, 9]. In a composite construction, such as the Charnley stem, there should be perfect bonding between stem and cement. In contrast, there is the construction in which the stem is not intended to be bonded to the cement, but rather to act as a “loaded taper” that is constrained by its surrounding cement mantle, such as the Exeter stem.

With uncemented fixation, primary stability is most important, as it facilitates osseointegration and thereby secondary stability.

Both uncemented stems and cemented loaded taper stems are meant to be somewhat “loose” shortly after surgery. A relatively high amount of migration is consequently expected when the patients start weight-bearing. In contrast, the composite construction should be mechanically stable after setting the cement. Only a small amount of migration is then to be expected during early postoperative rehabilitation.

When levels of early migration are expected to vary due to differences in fixation, implant design, etc., it could be wise to disregard early migration data so as to make study comparisons more comprehensive. Early postoperative RSA measurements typically serve as a baseline for measures of implant movement. To overcome expected differences in early implant movements, 2 or 3 years of data have been suggested for use as the baseline when comparing levels of migration [10].

The rationale of predicting medium- to long-term prosthetic loosening based on short-term migration data is based on publications that focus on different implants and different measurement techniques [6, 7, 11, 12].

Implant-specific migration data could be useful to improve the prediction of late implant loosening based on short-term migration data. Background data from well-functional, nonrevised total hip replacements are then needed to establish acceptable limits of femoral stem migration. In the present RSA study, we describe the migration pattern of the Charnley hip stem in a group of well-functioning patients at 5-year follow-up.

## Materials and methods

The study was approved by the Norwegian Technical and Scientific University Central Region Ethics Committee (reference 094-02), and conducted in accordance with the Declaration of Helsinki.

Originally, this study was designed as a 2-year prospective randomized cohort RSA study to investigate the stability of the Charnley flange 40 femoral stem prosthesis fixated with either Palacos R or SmartSet HV cement. No significant difference in stem fixation was found at the 2-year follow-up [13]. According to the clinical evaluation, all patients were performing very well clinically and the study was extended to include a 5-year follow-up. The extension was approved by the Ethics Committee. The main purpose of the study was then to describe the migration pattern of the Charnley stem, regardless of the type of cement employed.

The patients were recruited from Trondheim University Hospital and operated on by one experienced surgeon (OSH). Thirty-five patients, representing 36 hips, were asked, and all agreed to participate. Informed patient consent was signed on the day of surgery.

Patients with an existing condition, such as malignancy, pregnancy, severe osteoporosis, corticosteroid treatment, disabling musculoskeletal problems (other than in the hips), as well as patients who had already participated in a clinical study with an investigational product within the last 6 months were not invited to participate. No exclusions were made based on these conditions.

The 23 patients were operated between October 2002 and October 2003. A bilateral procedure where both hips received the Palacos cement was performed in one patient. Mean (range) age and mean (range) weight at surgery were 69 (60–76) years and 75 (60–98) kg, respectively. At five-year follow-up 22 patients were diagnosed with osteoarthritis and one patient with congenital hip dysplasia.

The cement randomization was performed preoperatively by a computer program (SAS version 8). An equal number of hips was allocated to each cement group. No stratification based on age or gender was made. The cement type to be used was revealed peroperatively. Differences in color and handling made the surgeon aware of the cement type allocated, while the patients were kept unaware.

**Fig. 1:**
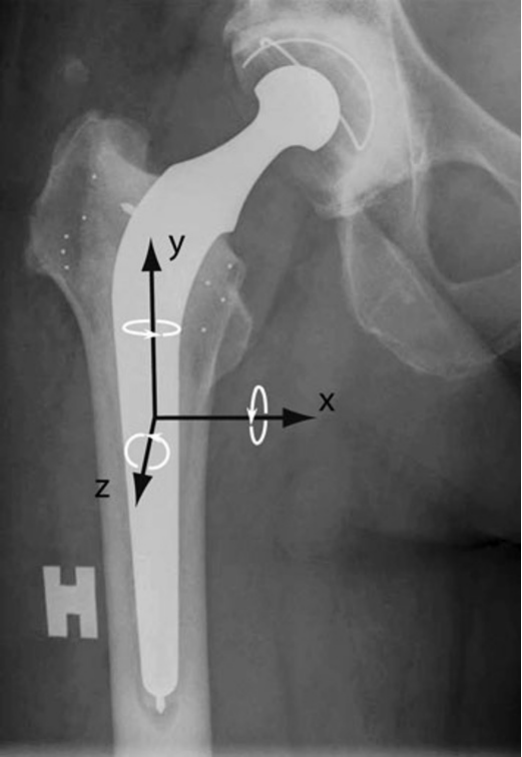
The coordinate system

The Charnley flanged 40 prosthesis (DePuy International, Leeds, UK) was implanted. This is a noncollared, shape-closed, stem with a proximal flange. The Charnley prosthesis was manufactured with tantalum beads mounted on stainless towers. One tower was fixed to the distal tip of the stem and one to the proximal shoulder. The central point of the prosthetic head served as the third marker (Fig. 1). All patients received the standard Charnley Ogee cup (DePuy International, Leeds, UK) with no embedded tantalum beads. A third-generation cementing technique incorporating retrograde filling and distal occlusion (Cement Restrictor, DePuy International) during pressurization was used for all patients. Palacos R was stored at 8 °C for a minimum of 24 h before use, while SmartSet HV was stored at 21 °C. All cements were mixed using a vacuum (Cemvac system, DePuy CMW, Blackpool, UK). For each patient, both the femoral and the acetabular components were fixated with the same type of cement.

A posterolateral surgical approach was used, with a lateral incision. The femoral canal was opened through the piriformis fossa and prepared with a 12 mm central reamer, followed by a 1 mm all-direction oversized broach. Intrafemoral pulsed lavage followed by a temporary 1 % adrenalin-soaked sponge were applied to prepare the cavity prior to cementing.

We performed the RSA follow-up according to the Selvik method [14]. Nine tantalum beads (0.8 mm in diameter) were implanted to serve as references: ideally five in the greater trochanter and four in the lesser trochanter region. The initial RSA examination was performed within 1 week postoperatively, after weight bearing (Table 1).

The RSA measurements were performed using UmRSA software (version 6.0, RSA Biomedical Innovation, Umea, Sweden). The tantalum balls had to be sufficiently scattered within each segment to ensure acceptable quality measurement. The condition number (CN) is a measure of the configuration of the markers in the segment. The mean error of rigid body fitting (ME) is a measure of marker stability. The upper limit on the ME was set to 0.25 mm, and the upper limit for the CN was set to 130, according to the guidelines in the*Instructional RSA User Course Manuala*(RSA Biomedical). The ME and CN were met in all analyses except for 1. This patient was excluded. The results for the present study are presented in Table 2.

Several RSA studies conclude that the migration of a cemented femoral stem mainly occurs at the implant–cement interface, not between the cement mantle and surrounding bone [15–17]. Tantalum markers that are mixed into the cement are difficult to scatter and also difficult to visualize on radiographs [17]. The present study was not designed to separate these two possible migrations, so all migration data refer to the movement of the implant in relation to the femoral bone.

It is recommended that the measurement precision in each particular study should be defined using double examinations [18]. We calculated the precision levels by performing 22 double examinations at the 2-year follow-up. Each patient rose from the X-ray table and walked between the two sequential examinations within 10 min (Table 5).

The right hand side was used as the reference for the coordinate system when defining the micromotions [18]. Thus, the*y* rotation,*z* rotation, and*x* translation values for the left hips were changed to relate to a right-handed coordinate system (Fig. 1).

The patients returned for follow-up at 3, 6, 12, 24, and 60 months. The numbers of RSA and clinical examinations at different time intervals are presented in Table 3. At each follow-up, the data from standard radiographs, RSA, and Merle d’Aubigné-Postel were recorded. The Merle d’Aubigne-Postel score (which measures pain, mobility, and ability to walk) has a maximum possible score of 18.

### Statistical methods

The independent samples two-tailed *t* test was used to analyze the differences in migration between the two cement groups at 5 years postoperatively.*Q*–*Q* plots were used to verify the normality of the distribution. The measurement data from the two groups were subsequently pooled, and descriptive data were calculated for the whole patient population.

Each precision level was calculated in two steps. First, the differences between the two examinations were calculated for each patient. Second, the standard deviation (SD) of these differences (with respect to zero) was calculated.

Here,*x* represents the differences between the double examinations, *n* = 22.

Finally, the precision was obtained by multiplying the SD by 2.074 (representing the 0.975 % point in a*t*_22_ distribution).

## Results

Twenty-three patients were examined at the 5-year follow-up. The Merle d’Aubigné-Postel score was completed for each of the 23 patients. The mean score and range were 18 (17–18) for all patients in both groups (Table 1). All 23 patients except 4 reached the maximum score. Three patients had value score 5 for pain, which indicates “pain is rare and mild”. One patient had value score 5 for mobility, indicating a mobility of 70°–90° [19].

**Table 1:** Patient demographic data

	At inclusion	At 5-year follow-up
Number of patients (hips)	35 (36)	23 (23)
Females:males	24:12	18:5
Merle d’Aubigné-Postel score, mean (range)	10 (7–13)	18 (17–18)
Reasons for not attending (*n*)		Deceased (4) Did not want to attend (4) Missed the RSA postop measurement (4) Condition number exceeded 130 (1)

**Table 2:** Mean error of rigid body fitting and condition number

	Femur	Implant
*Mean error of rigid body fitting* (*ME*)		
Mean	0.17	0.08
Range	0.10–0.24	0.03–0.21
*Condition number* (*CN*)		
Mean	56	34
Range	31–115	34–35
*Markers visible*		
Mean	6	4
Range	3–8	4–4

**Table 3:** Number of RSAs and clinical examinations at different time intervals

Follow-up time	Number of examinations
Postop	32
3 months	27
6 months	20
12 months	32
24 months (precision)	28 (22)
60 months	23

Number in parenthesis represents the number of double examinations

Examination of the 5-year follow-up conventional radiographs showed no osteolysis or radiological signs of loosening.

First, the data from both cement groups were compared. No statistically significant differences in migration were observed between the two groups at 5 years postoperatively (Table 4).

**Table 4:** Migration data at the 5-year follow-up (mean values) along with corresponding *p* values for the two-tailed independent samples *t* test comparing the two cements

Axes	Rotation (°)			Translation (mm)		
	*x*	*y*	*z*	*x*	*y*	*z*
SmartSet HV	−0.08	1.39	−0.23	0.00	−0.19	−0.05
Palacos R	−0.17	1.90	−0.18	−0.03	−0.22	−0.24
*p* values	0.61	0.10	0.75	0.45	0.12	0.12

**Fig. 2:**
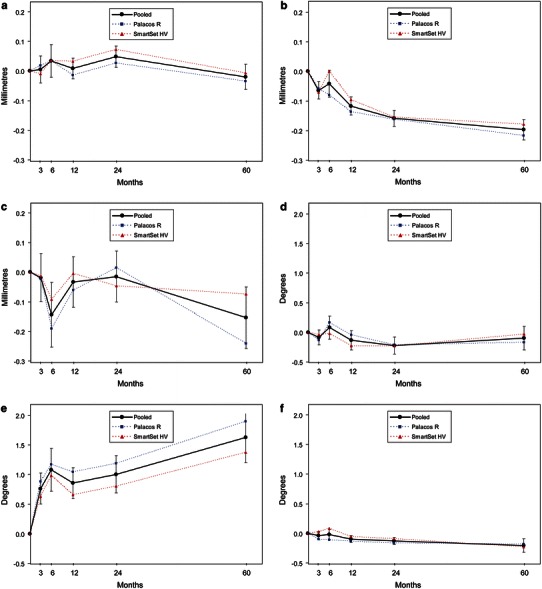
**a** Translation along the*x*-axis. The graph represents the mean and 95 % confidence interval (CI) for the pooled data. **b** Translation along the*y*-axis. The graph represents the mean and 95 % confidence interval (CI) for the pooled data. **c** Translation along the*z*-axis. The graph represents the mean and 95 % confidence interval (CI) for the pooled data. **d** Rotation around the*x*-axis. The graph represents the mean and 95 % confidence interval (CI) for the pooled data. **e** Rotation around the*y*-axis. The graph represents the mean and 95 % confidence interval (CI) for the pooled data. **f** Rotation around the*z*-axis. The graph represents the mean and 95 % confidence interval (CI) for the pooled data

The data from the two cement groups were then pooled. The migration measurement results for the merged data are presented in Fig. 2a–f. The measurement data were found to be normally distributed for all 6 data sets.

## Discussion

This follow-up study describes the migration pattern of the Charnley flanged 40 femoral stem. A total of 35 patients (36 hips) were enrolled, and data from 23 patients (23 hips) at 5 years were obtained. The patients were followed and evaluated by RSA as well as by clinical examination. The Merle d’Aubigné-Postel score at 5 years indicated that the patient group was functioning well. None of the patients lost to follow-up had their hips revised.

Originally the study was designed to compare stem stability upon using SmartSet HV and Palacos R bone cement in a prospective randomised 2-year follow-up study. No differences in stem stability were found [13]. In the present study at 5-year follow-up, both cements also performed equally well. The most striking finding at this point was the observation of the continuous migration of a composite construction. Stem subsidence (translation along the*y*-axis) and stem retroversion (rotation around the*y*-axis) continued progressively up to 5 years postoperatively. Consequently, the main focus was then to describe the overall stem migration at 5 years regardless of the cement used.

The precision levels found in the present study (Table 5) are comparable to those seen in other studies—somewhat poorer than those presented by Hallan et al. [16], but somewhat better than those presented by Onsten et al. [20].

**Table 5:** Precision of the RSA measurements

Axes	Rotation			Translation		
	*x*	*y*	*z*	*x*	*y*	*z*
Precision	0.43	1.01	0.12	0.10	0.10	0.22

As seen in Table 5, the precision levels vary considerably between the 3 rotational migrations measured. The tantalum markers mounted on the implant define a polygon. Evaluating the scattering of the markers is vital in order to determine the migration of the implant (polygon) in relation to the rotational axes. The method is more sensitive to rotations when the markers are located far from the rotational axes. Good scattering of the markers improves the level of precision accordingly. This phenomenon is generally seen in RSA measurements of hip implants.

Our results show that most of the rotations and translations take place initially. The translation along and the rotation around the*y*-axis represent stem subsidence and retroversion, respectively (Fig. 2b, e). The resultant joint force that is transferred by the prosthetic head and acts on the stem will tend to push the stem distally and rotate the stem internally inside the cement mantle. Consequently, the migration pattern presented herein should be expected. The results presented in Fig. 2b, e indicate persistent stem migration for both subsidence and internal rotation. Even though the subsidence is minor, it indicates persistent migration. The rotation into varus also increases, and was 0.18° at 5 years postoperatively (Fig. 2f).

All 6 migrations at 5 years in the present study are equal to or smaller than those noted in the 2-year follow-up Charnley study by Hallan et al. [16] (a longer follow-up period is not reported). Our 5-year data confirm this inclination.

We found the 5-year translation values to be considerably lower than what Onsten et al. described in a 2-year follow-up of 65 Charnley stems, where most of the migration took place during the first 3 months. Mean migration along the*y*-axis was 0.24 mm, along the*x*-axis 0.31 mm, and along the*z*-axis 0.67 mm. The rotations were not reported [20].

Different stem designs are expected to migrate differently [8, 15]. Kärrholm et al. [6] believe that the quantity of subsidence after 2 years is the best predictor of medium- to long-term loosening. Subsidence of the femoral head of 1–2 mm during the first 2 postoperative years indicated an increased risk of loosening and revision. The postoperative examinations were performed over a time interval stretching from the first day to 2 months after surgery. The patient population consisted of both primary THR and revisions. The implant was the cemented Lubinus SP I prosthesis [6]. Several factors—type of implant, follow-up time, time for baseline RSA, and precision level—mean that Kärrholm et al.’s study and results are not directly comparable to those from our study.

Ryd et al. argues that a migration exceeding 0.2 mm at 2 years implies an increased risk of implant loosening. This study on knees evaluated several brands of (cemented and uncemented) knee prostheses. Mechanical loosening occurred exclusively in prostheses which migrated continuously [7]. Our study is not straightforward comparable to their study, since there are fundamental differences between hip and knee replacement, and Ryd et al. used maximum total point motion (MTPM) to identify migration, while we used orthogonal translations and rotations to describe segment motions.

The study of Ryd et al. dealt with knee prostheses, while that of Kärrholm et al. evaluated primary and revision hips. This may explain the large disparity between their two different limits for increased risk of loosening, which differ by factors of 10, 1–2 mm, and 0.2 mm in the two studies, respectively [6, 7]. This exemplifies the importance of more implant-specific migration data that can be used to predict long-term implant survival based on short-term migration data.

The Exeter stem is constructed as a loaded, double-tapered, collarless, polished stem, and is designed to subside, in contrast to the Charnley stem [15]. A study with the Exeter stem found a mean subsidence of 1.25 mm at the 2-year follow-up. The mean internal rotation was 0.6° and the varus alignment was 0.21° [21]. Another follow-up study of the Exeter stem showed continuing subsidence (1.77 mm, median) and retroversion (1.6°, median) up to 5 years. This may be expected with this type of fixation [17].

The Charnley stem is not supposed to migrate but to show immediate stability due to its composite construction [15]. In spite of this, studies have confirmed that the Charnley stem does migrate and rotate [13, 16, 20]. Our study supports these findings and also demonstrates that migration continues until at least 5 years.

A number of earlier RSA studies on hips differ from current guidelines in the standardization of RSA investigation. The different measuring methods, follow-up intervals, and type of implants used constitute an obstacle to any comparison of these heterogeneous results. Planned RSA studies ought to act in accordance with published guidelines for the standardization of RSA [18] so that it is easier to compare them.

Since several factors appear to influence the level of migration, it may be difficult to define acceptable limits on implant short-term migration with respect to long-term survival [4]. More implant-specific RSA background data are needed to predict what can be considered to be acceptable micromotion. Migration data are continuous.

In conclusion, we have found that the Charnley stem continued to subside and to rotate in retroversion up to 5 years after surgery in a well-functioning patient group. More implant-specific RSA data are needed to predict long-time implant survival based on short-term observations. The migration data presented herein could contribute to this, serving as reference data for a normal migration pattern of a Charnley flanged 40 prosthesis.
